# Approaching precision public health by automated syndromic surveillance in communities

**DOI:** 10.1371/journal.pone.0254479

**Published:** 2021-08-06

**Authors:** Ta-Chien Chan, Jia-Hong Tang, Cheng-Yu Hsieh, Kevin J. Chen, Tsan-Hua Yu, Yu-Ting Tsai

**Affiliations:** 1 Research Center for Humanities and Social Sciences, Academia Sinica, Taipei, Taiwan; 2 Institute of Public Health, School of Medicine, Yang-Ming Chiao Tung University, Taipei, Taiwan; 3 Institute of Statistical Science, Academia Sinica, Taipei, Taiwan; 4 Department of Health, Taipei City Government, Taipei, Taiwan; The Chinese University of Hong Kong, HONG KONG

## Abstract

**Background:**

Sentinel physician surveillance in communities has played an important role in detecting early signs of epidemics. The traditional approach is to let the primary care physician voluntarily and actively report diseases to the health department on a weekly basis. However, this is labor-intensive work, and the spatio-temporal resolution of the surveillance data is not precise at all. In this study, we built up a clinic-based enhanced sentinel surveillance system named “Sentinel plus” which was designed for sentinel clinics and community hospitals to monitor 23 kinds of syndromic groups in Taipei City, Taiwan. The definitions of those syndromic groups were based on ICD-10 diagnoses from physicians.

**Methods:**

Daily ICD-10 counts of two syndromic groups including ILI and EV-like syndromes in Taipei City were extracted from Sentinel plus. A negative binomial regression model was used to couple with lag structure functions to examine the short-term association between ICD counts and meteorological variables. After fitting the negative binomial regression model, residuals were further rescaled to Pearson residuals. We then monitored these daily standardized Pearson residuals for any aberrations from July 2018 to October 2019.

**Results:**

The results showed that daily average temperature was significantly negatively associated with numbers of ILI syndromes. The ozone and PM_2.5_ concentrations were significantly positively associated with ILI syndromes. In addition, daily minimum temperature, and the ozone and PM_2.5_ concentrations were significantly negatively associated with the EV-like syndromes. The aberrational signals detected from clinics for ILI and EV-like syndromes were earlier than the epidemic period based on outpatient surveillance defined by the Taiwan CDC.

**Conclusions:**

This system not only provides warning signals to the local health department for managing the risks but also reminds medical practitioners to be vigilant toward susceptible patients. The near real-time surveillance can help decision makers evaluate their policy on a timely basis.

## Introduction

Sentinel physician surveillance is a traditional and fundamental approach to monitor disease activity in communities. Many countries such as France, Japan, and the United Kingdom have set up sentinel-based surveillance for a long time [1–3]. Different countries have had different focuses of diseases, but influenza-like illness (ILI) has been one of the common focuses for surveillance. The data from sentinel surveillance can be used to compute the incidence of diseases or outpatient consultation rate [4]. In order to cope with emerging infectious diseases or the potential threats of bioterrorism, syndromic surveillance in the emergency room (ER) has been developed to detect any aberrations of syndromic groups rather than specific diseases [5]. The success of using such a surveillance system has in part been due to the fact that it does not need any extra work from physicians. The monitored data are automatically retrieved and computed from hospital information systems (HIS). The algorithms for aberration detection can be applied to either disease diagnosis codes based on the International Classification of Diseases Ninth or Tenth Revision (ICD-9 or ICD-10) [6] or free-text-based chief complaints [7]. Although surveillance in an ER setting has shown good performance for early warning, our past experience showed that aberration signals of ILI from an outpatient setting can be earlier than those from ER, especially for novel pandemic flu in 2009 [8]. In the United States, the feasibility of applying outpatient surveillance for ILI at the community level has been demonstrated, with a pattern consistent with syndromic and virological surveillance [9]. The advantages of syndromic surveillance and sentinel surveillance can be integrated to ensure community health quickly and precisely. The idea of precision public health can take the right intervention for the right population swiftly [10]. The United Kingdom has had national syndromic surveillance based on multiple health care settings including a telehealth triage system, general practice and ER for 20 years [11]. The system has become an important component of the public health system for early warning and situational awareness.

In Taiwan, there have been many infectious-disease-related surveillance systems (https://nidss.cdc.gov.tw/en/) such as notifiable infectious disease reporting systems, school-based infectious disease reporting systems, syndromic surveillance systems in ER, and outpatient and hospitalization surveillance from national health insurance data. In December 2009, Taiwan’s CDC (Centers for Disease Control) discontinued its sentinel physician surveillance system for two major reasons, including the labor-intensive reporting process (86.08% reporting via fax or phone) and the representativeness issue [12]. Syndromic surveillance and national health surveillance can capture the epidemic situation at a national or city level, but the timeliness of data exchange, spatial resolution and the number of monitored syndromic groups are limited. Based on past epidemic experience, daily and small-area surveillance can detect early aberrations [8]. In addition, emerging infectious diseases do not have typical symptoms at the early stage of an epidemic. Traditional disease-based reporting systems cannot capture this kind of signal. Therefore, we set up a clinic-based surveillance system to monitor 23 kinds of syndromic groups in communities. Through longitudinal surveillance and sensitive statistical models, the system can automatically remind medical practitioners of the epidemic situation of different syndromic groups and help them remain vigilant to susceptible patients. Furthermore, local health departments can take action based on aberrations to prevent the epidemic from getting worse and to reduce the severity of the infected cases. In this study, we used our first-year data on ILI and enterovirus-like (EV-like) illness in Taipei City, Taiwan to evaluate the timeliness of the “Sentinel plus” system for detection of aberrations at the earliest stages.

## Materials and methods

### Ethics

This study was approved by the Institutional Review Board on Biomedical Science Research, Academia Sinica (AS-IRB-BM-18017). For participating clinics, the research team needed to receive written consent from the chief physician who is in charge of the clinic first and then activated their accounts. The collaborating two companies in charge of the clinics’ hospital information systems (HIS) helped set up the computing and uploading a plugin in the systems’ background. The data collected in these systems were all aggregated data without any patient identifiers. The data were aggregated into 23 syndromic groups and seven age groups before being uploaded to the HIS providers’ server. The data visualization is displayed with four different spatial resolutions including clinics, villages, townships and the whole city. The ILI and EV-like illness aggregated data we used are provided at the open repository, figshare.com (10.6084/m9.figshare.11497137).

### Data source

**Fig 1:**
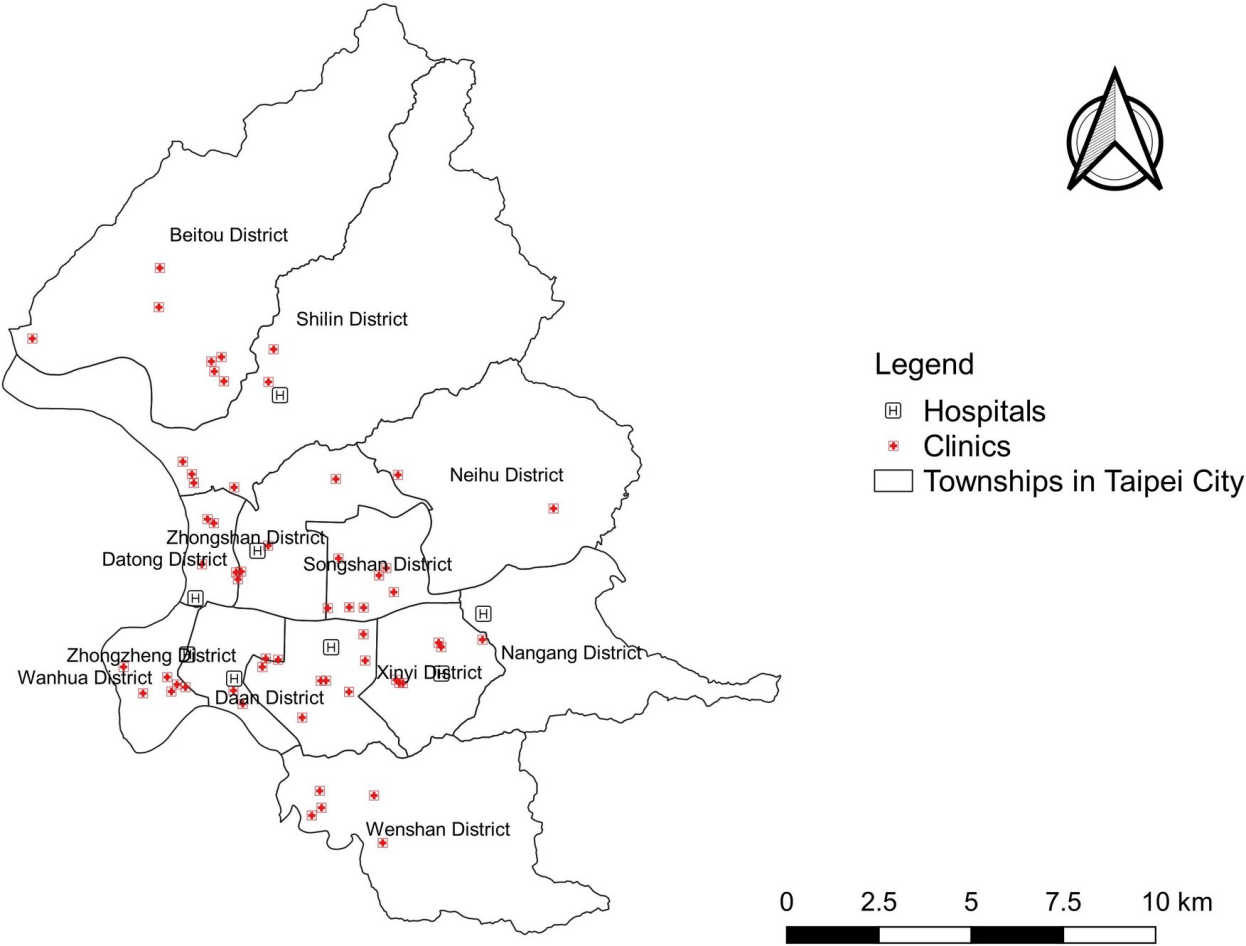
Spatial distribution of participating clinics and community hospitals in Taipei City.

In July 2018, we began to set up a clinic-based enhanced sentinel surveillance system named “Sentinel plus” which was designed for sentinel clinics and community hospitals to monitor 23 kinds of syndromic groups to detect early signs of epidemics. The list of the 23 syndromic groups is shown in S1 File. A snapshot of the Sentinel plus is shown in S2 File. Due to the closed environment of information systems in clinics, they used different HIS and data exchange via virtual private networks (VPN). The research team collaborated with the HIS providers with the top two market shares in Taiwan to pipeline aggregated data from clinics to Sentinel plus. In addition, the data from community hospitals were via point-to-point transmission with encryption. The specific International Classification of Diseases (ICD) codes of monitored syndromic groups from medical records of participating clinics were computed. Here, we used two syndromic groups, i.e. ILI for all ages and EV-like illness for ages 0–6 from Sentinel plus. ICD-10 diagnoses of daily visits from July 2018 to July 2019 were obtained from 58 participating clinics and eight community hospitals in Taipei City (Fig 1). EV-like data were not collected in the community hospitals. Thus, the EV-like data only include data from clinics.

The definition of ILI with ICD-10 diagnoses was adopted from the International Society for Disease Surveillance, and the definition of EV-like illness was adopted from one local study [13] in Taiwan. In addition to surveillance data, we also incorporated weather data and air pollutants into the prediction model. Daily meteorological data were downloaded from Taiwan’s Central Weather Bureau (https://e-service.cwb.gov.tw/HistoryDataQuery/index.jsp). Air quality monitoring data were downloaded from the Taiwan Air Quality Monitoring Network (TAQMN) (https://taqm.epa.gov.tw/taqm/en/YearlyDataDownload.aspx).

### A general representation of the statistical model

The relationship between environmental exposure and health outcomes, such as commonly daily mortality or morbidity counts, is an important research topic, and many epidemiological studies attempt to elucidate these links [14, 15]. However, count data are often overly dispersed. A common way to deal with overdispersion for count data is to use a generalized linear model (GLM) framework, where the most common approach is a quasi-Poisson or a negative binomial regression model [16]. Moreover, the effect of a specific environmental exposure event is not limited to the period when it is observed, but is delayed in time [17, 18]. This situation also occurs when assessing the short-term effects of environmental stressors, such as meteorological conditions [19, 20] and air quality [21, 22], for ILI and EV. Therefore, it requires the use of statistical models that are flexible enough to describe lagged associations between the health outcomes and environmental factors. Steps in variable selection during model building are described as follows.

Step 1. Reduce the number of variables in the dataset by selecting candidate variables for the model. This can be achieved by reviewing the existing literature and consulting with experts.

Step 2. Apply appropriate variable selection method within the candidate variables to include variables in the final model.

If we are interested in finding to what extent there is a numerical relationship between a response variable and explanatory variables of interest, using their correlation coefficients will give misleading results if there is another, confounding variable that is numerically related to both variables of interest. This misleading information can be avoided by controlling for the confounding variables, which is done by computing the partial correlation coefficient. To decide whether or not a covariate should be added to the model, the partial correlation test, which controls the effect of other collected covariates, was conducted in this study. Moreover, variance inflation factors were used for testing multicollinearity to avoid covariates being correlated.

In this study, data on ICD-10 diagnoses come in the form of counts, and we relate these counts to environmental factors and other covariates. To better account for over-dispersion and lagged dependencies, we used a negative binomial regression model coupled with lag structure functions to examine the short-term association (day-to-day variation) between ICD counts and *k* meteorological variables. This model represents the counts of outcome events *y*_*t*_ at day *t* as

$$\log\left(E(y_{t})\right)=\log(\Delta )+\beta_{0}+\sum_{j=1}^{m}\alpha_{j}∙s_{j}(x_{j},\;w_{j};\;t)+\sum_{k=1}^{p}\beta_{k}z_{k,\;t},\;t=1,2,\ldots ,n$$ (1)

where *α*_*j*_, *j* = 1,2,…,*m*, *β*_0_, and *β*_*k*_, *k* = 1,2,…,*p* are regression coefficients. The logarithm of Δ is an offset term. In this study, the number of clinics was specified as an offset variable. The number of clinics here refers to the number of clinics that report ICD codes of a certain disease on the day. Although most clinics are closed on Sundays and public holidays, the opening days during weekdays may vary among clinics. Furthermore, not every participating clinic will have patients with a certain disease every day, such as ILI or EV-like disease. Thus, the offset term in the model is used to correct for the variation in the number of clinics. The variables *z*_*k*,*t*_ include other covariates with linear effects specified by the related coefficients *β*_*k*_. Statistical significance for model coefficients was 0.05. The function *s*_*j*_ and two row vectors $x_{j}=(x_{j,\;t‐l_{0}},\ldots ,x_{j,\;t‐l},\ldots ,x_{j,\;t‐L})$, $w_{j}=(w_{j,\;t‐l_{0}},\ldots ,w_{j,\;t‐l},\ldots ,w_{j,\;t‐L})$ will be described in detail later.

To explore the influence of environmental factors on ICD-10 counts of the ILI and EV-like syndromic groups, two meteorological factors—average temperature for ILI and minimum temperature for EV-like illness and relative humidity—and two air pollutants—PM_2.5_ and ozone—are considered in the proposed model. Considering the collinearity and the goodness of fit, different temperature variables were used in building the model for different syndromes in this study. Furthermore, two additional covariates—day of the week and public holidays—are included as dummy variables in both models, so as to cope with calendar effects.

### Modelling lagged associations

In the presence of delayed effects for ***x***_*j*_, the outcome at a given time *t* may be explained in terms of past exposures *x*_*j*, *t-l*_, with *l* as the lag, representing the period elapsed between the exposure and the response. The main complexity of modeling and interpreting dependencies lies in the additional temporal dimension needed to express the association, beyond the usual exposure–response relationship, as the health outcome depends on both intensity and timing of past exposure. Nonetheless, the appropriate representation of the temporal pattern of such dependencies may provide further insights into the association of interest and prevent biases in estimates and predictions.

Assuming a linear exposure–response relationship, a general notation of exposure–response function, *s*_*j*_, for ***x***_*j*_ can be given by

$$s_{j}(x_{j},\;w_{j};\;t)=x_{j}w_{j}^{T}/\sum_{l=l_{0}}^{L}w_{j,\;t‐l}=(\sum_{l=l_{0}}^{L}x_{j,\;t‐l}∙w_{j,t‐l})/\sum_{l=l_{0}}^{L}w_{j,\;t‐l}$$ (2)

where $x_{j}=(x_{j,\;t‐l_{0}},\ldots ,x_{j,\;t‐l},\ldots ,x_{j,\;t‐L})$ and $w_{j}=(w_{j,\;t‐l_{0}},\ldots ,w_{j,\;t‐l},\ldots ,w_{j,\;t‐L})$.

The functions *s*_*j*_ denote smoothed relationships defined by the lag-response vector ***w***_*j*_ in terms of the exposure history to the environmental factor ***x***_*j*_ measured over the lag interval *l* = *l*_0_, …, *L*, with *l*_0_ and *L* as the minimum and the maximum lag, respectively. This parameterization is such that the lag-response function *w*_*j*, *t-l*_ is directly defined as a lag structure which may represent different time scales depending on the study.

In this study, the lag-response function *w*_*j*, *t-l*_ is modeled by indicators for each lag *l* to identify the linear exposure–response association and is defined as: *w*_*j*, *t-l*_ = 1, if the partial correlation between *x*_*j*, *t-l*_ and *y*_*t*_ is statistically significant; and *w*_*j*, *t-l*_ = 0, otherwise. Statistical significance for the partial correlation was 0.1.

### Aberration detection rule and forecasting procedure

After fitting the negative binomial regression model, residuals were further rescaled to Pearson residuals [8]. The standardized Pearson residuals from the fitted model on *n* consecutive days were denoted as

$$R_{t}=\left(y_{t}‐{\hat{y}}_{t}\right)/\sqrt{\mathrm{Var}({\hat{y}}_{t})},\;for\;t=1,2,\ldots ,n,$$ (3)

where ${\hat{y}}_{t}$ is the fitted value given by the model. We then monitored these daily standardized Pearson residuals for any aberrations.

Since the sample size *n* is often large, the standardized Pearson residuals could be assumed to be approximately distributed normally, with mean 0 and variance 1. Therefore, we proposed a simple rule by directly monitoring the series of Pearson residuals. When a Pearson residual was larger than the 100(1−*α*)th percentile of the standard normal distribution, a signal was issued for the day to report a possible aberrant outbreak. In this study, the 97.5th percentile of the standard normal distribution, i.e. 1.96, was used to set the threshold.

A main purpose of syndromic surveillance systems is usually to detect disease outbreaks rapidly and effectively for guiding interventions to control epidemics. Thus predictability is one of fundamental components of syndromic surveillance systems. To evaluate stability and predictive accuracy, a rolling analysis with a fixed window is used to back-test our model on the data in this study. The visiting volume and the age structure of the patients are different in clinics and hospitals. Therefore, we separated the two kinds of data for model fitting and prediction. In the clinics’ part, the count data of a total of 475 consecutive days (from 14 July 2018 to 31 October 2019) are then split into a training set (from 14 July 2018 to 30 June 2019) and a testing set (from 1 July 2019 to 31 October 2019). The training set consists of the first 352 data records, and the testing set consists of the rest of the data. We use the rolling window method to keep the sample size constant at 352, by adding the 353th observation and dropping the first observation. This procedure is repeated until the last observation of the entire sample. In the community hospitals’ part, the count data of a total of 473 consecutive days (from 16 July 2018 to 31 October 2019) collected are also split into a training set (from 16 July 2018 to 30 June 2019) and a testing set (from 1 July 2019 to 31 October 2019). The constant rolling window of HIS data is set to 350 for the rolling window method.

The rolling forecast errors for the one-step-ahead forecast are computed across all testing data, and predictive performance is evaluated using the root forecast mean squared error (RMSE) and the mean absolute percentage error (MAPE), which are given as follows.


$$\mathrm{RMSE}=\sqrt{\frac{1}{n}\sum_{t=1}^{n}{\left(y_{t}‐{\hat{y}}_{t}\right)}^{2}}$$ (4)

$$\mathrm{MAPE}=(\frac{1}{n}\sum_{t=1}^{n}\frac{\left|y_{t}‐{\hat{y}}_{t}\right|}{y_{t}})\times 100\%$$ (5)

In addition, comparison with the reference standard was required to evaluate the performance of early aberration detection alerts from clinics and community hospitals, respectively. Although it was difficult to find a gold standard for defining the epidemic period, we use an intuitive method to establish the "ground truth" about the existence and timing of an epidemic. Two types of reference datasets were collected from open data of the Taiwan CDC, including the syndromic surveillance system in ER, and outpatient surveillance from national health insurance data, respectively. The temporal resolution of the data is weekly, starting from Sunday to Saturday. The spatial resolutions of outpatient and ER data are different because of the availability of the total volume of visits. In outpatient data, the spatial unit is Taipei City. In ER data, the spatial unit is the Taipei region including Taipei City, New Taipei City, Keelung City, Yilan County, Lianjiang County, and Kinmen County. Two standard deviations above the mean during the study period was used as the threshold of the gold standard method for both datasets to determine when an epidemic occurred. Aberration signals were plotted on Saturday when the number of weekly reported cases in the corresponding week exceeding the defined threshold.

## Results

Summary statistics of environmental factors over the training period are presented in Table 1. It should be noted that the temperature variable was different in ILI and EV-like syndromes because of the model selection. Daily average temperature was used for daily ILI syndromic surveillance, while the daily minimum temperature was used for daily EV syndromic surveillance.

**Table 1:** Descriptive statistics for daily data of environmental factors and ICD-10 counts of ILI and EV in Taipei City.

Environmental factor (unit)	Min	Q_1_	Q_2_	Q_3_	Max	Mean	Standard deviation
Temperature (°C) (daily average)	13.30	19.98	23.45	28.10	31.90	23.55	4.74
Temperature (°C) *(*daily minimum*)*	11.90	17.48	20.90	25.13	28.70	21.06	4.35
Relative	50.30	68.71	76.00	82.70	93.70	75.24	9.11
humidity (%)							
O_3_ (ppm)	10.00	33.61	41.30	50.40	84.30	41.52	13.19
PM_2.5_ (μg/m^3^)	4.00	11.01	14.00	19.00	42.00	15.58	6.90
ICD-10 counts of ILI syndrome	49.00	803.50	1695.00	3200.75	5999.00	2071.07	1372.97
ICD-10 counts of EV syndrome	0.00	5.00	9.00	16.00	56.00	11.07	8.27

Different lag times of environmental factors were considered in the process of model selection. Table 2 shows the lag structure of each environmental factor for daily ILI and EV syndromic surveillance, respectively. In this study, the lag interval for each environmental factor was specified from one to seven days. Under the assumption of a linear exposure–response relationship, the lag-response function is the moving average of lagged exposures. For daily ILI syndromic surveillance, daily average temperature, relative humidity, concentration of ozone and concentration of PM_2.5_ were calculated using the moving average of lags 1–7, lag 2, lags 1 and 4, and lags 5–7, respectively. For daily EV syndromic surveillance, daily minimum temperature, relative humidity, ozone and PM_2.5_ concentrations were calculated using the moving average of lags 1–7, lags 2–3, lags 2–4 and 6, and lag 1, respectively.

**Table 2:** The lag-response functions of environmental factors for daily ILI syndromic surveillance.

	Environmental factor	Lag 1	Lag 2	Lag 3	Lag 4	Lag 5	Lag 6	Lag 7
ILI syndrome	Temperature (daily average)	1	1	1	1	1	1	1
	Relative humidity	0	1	0	0	0	0	0
	O_3_	1	0	0	1	0	0	0
	PM_2.5_	0	0	0	0	1	1	1
EV syndrome	Temperature (daily minimum)	1	1	1	1	1	1	1
	Relative humidity	0	1	1	0	0	0	0
	O_3_	0	1	1	1	0	1	0
	PM_2.5_	1	0	0	0	0	0	0

Results of the negative binomial regression model on the daily series of ILI and EV-like syndromes are presented in Table 3. In summary, the volume of ILI syndromes was significantly high on Mondays and low on Tuesdays, public holidays and days excluding not only public holidays but also the day after public holidays. Daily average temperature was significantly negatively associated with ILI syndromes (p<0.01). The ozone and PM_2.5_ concentrations were significantly positively associated with ILI syndromes. For EV surveillance, EV-like syndromes were significantly high on Mondays and Fridays. Daily minimum temperature, the ozone and PM_2.5_ concentrations were significantly negatively associated with the EV-like syndromes. During the stage of model fitting, the adjusted R-squared values for the ILI syndromic model and the EV syndromic model are 0.8867 and 0.7399, respectively.

**Table 3:** Estimated coefficients from negative binomial regression.

	ILI syndrome		EV syndrome	
	Estimate	Std. Error	Estimate	Std. Error
(Intercept)	4.7623**	0.1371	1.4513**	0.3094
Days of the week				
Monday	0.2874**	0.0347	0.2008**	0.0683
Tuesday	-0.1018**	0.0341	0.0676	0.0713
Wednesday	-0.0615*	0.0342	0.0102	0.0717
Thursday	-0.0506	0.0342	0.0039	0.0714
Friday	0.0151	0.0345	0.1530*	0.0719
Saturday	(Baseline)		(Baseline)	
Sunday	0.0213	0.0343	0.0816	0.0823
Public holidays				
Public holidays	-0.2579**	0.0752	-0.0662	0.1610
Day after public holidays	(Baseline)		(Baseline)	
Days excluding public holidays and day after public holidays	-0.1468*	0.0618	-0.0012	0.1208
Temperature (ILI: daily average, EV: daily minimum)	-0.0261**	0.0022	-0.0181**	0.0049
Relative humidity	-0.0009	0.0011	-0.0032	0.0025
O_3_	0.0019**	0.0009	-0.0094**	0.0022
PM_2.5_	0.0037*	0.0018	-0.0069*	0.0030

Note: ‘*’: p-value < 0.05

‘**’: p-value < 0.01

To test multicollinearity, we examined variance inflation factors (VIF) for both models including all explanatory variables. In S3 File, the VIFs are all less than 10, indicating that multicollinearity is not a serious concern.

**Fig 2:**
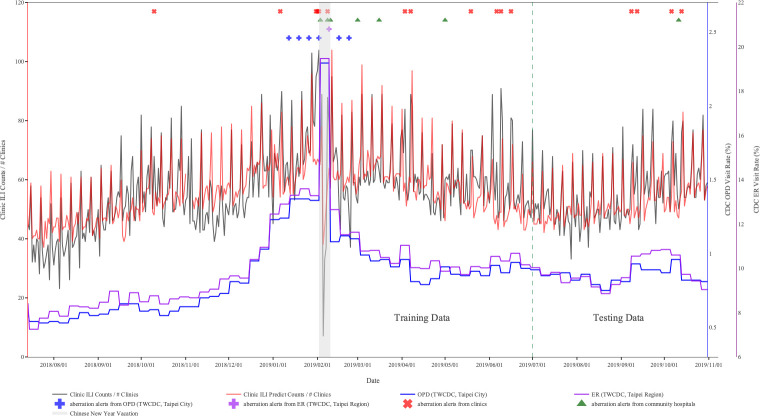
Results of aberration detection by proposed method using ILI syndromes collected from Sentinel plus.

We evaluated the performance of the proposed models for ILI and EV syndromic surveillance in aberration detection from 14 July 2018 to 30 June 2019 in the following, respectively. Fig 2 displays the time series of the average ILI syndromes from Sentinel plus. It also presents epidemic signals generated by two defined thresholds, and aberration alerts from clinics and community hospitals using our proposed model. Based on surveillance data of Taiwan’s CDC, there was an ILI epidemic period during January to February in 2019 and the first outbreak signal among outpatients (January 12, 2019; with blue + symbol) appeared earlier than that in ERs (February 9, 2019; with purple + symbol). For the results of our proposed model, the first aberration alert from clinics (January 6, 2019; with red X symbol) was triggered earlier than that from community hospitals (February 3, 2019; with symbol, green triangles). The first aberration alert of ILI from clinics appeared approximately six days earlier than the defined epidemic period from outpatient surveillance of Taiwan’s CDC, 28 days earlier than the first signal detected from community hospitals, and 34 days earlier than the first signal detected from ER surveillance of Taiwan’s CDC.

**Fig 3:**
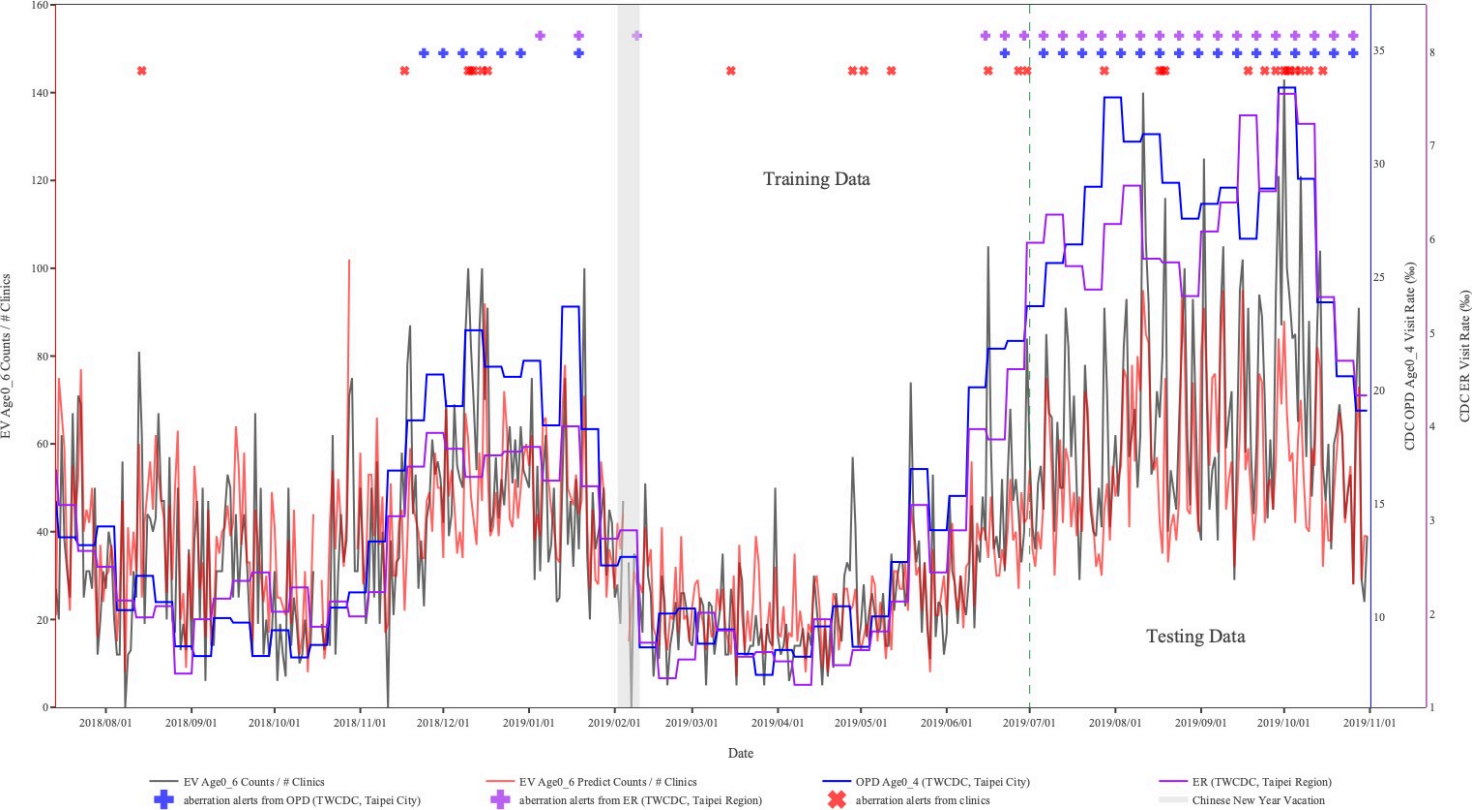
Results of aberration detection by proposed method using EV syndromes collected from Sentinel plus.

The EV-like data were only collected in clinics. Fig 3 demonstrates the time series of the average counts of EV-like syndromes from clinics. Based on outpatient surveillance of Taiwan’s CDC and the defined threshold in this study, there were two EV epidemics during the study period. One was from November 2018 to January 2019; the other was in June 2019. In the first epidemic period, the first aberration alert from clinics was generated on November 17, 2018 (with red X symbol) and was seven days earlier than the first outbreak signal of CDC EV outpatient surveillance (November 24, 2018; with blue + symbol) and 49 days earlier than CDC EV ER surveillance (January 5, 2019; with purple + symbol). In the second EV epidemic period, the first aberration alert from clinics was issued on June 16, 2019 (with red X symbol). Compared to the two streams of EV surveillance from Taiwan CDC, the first aberration alert of clinics was six days earlier than the CDC EV outpatient surveillance (with blue + symbol) but one day later than the CDC EV ER surveillance (with purple + symbol).

Fig 2 also shows that the proposed model issued sporadic alerts during the test period of the ILI. However, there was no alert issued by OPD and ER surveillance. In Fig 3, the proposed model based on ICD counts first shows the first aberration alert in mid-June during the test period (1 July 2019 to 31 October 2019) of EV-like syndrome. Compared with the open weekly data for OPD and ER released by Taiwan’s CDC, the proposed model can issue alerts one week or the first day of the week before the related peaks. By contrast, based on OPD and ER surveillance of Taiwan’s CDC and the defined threshold in this study, their data generated too many false alerts, with both of them generating alerts weekly from August to October.

**Fig 4:**
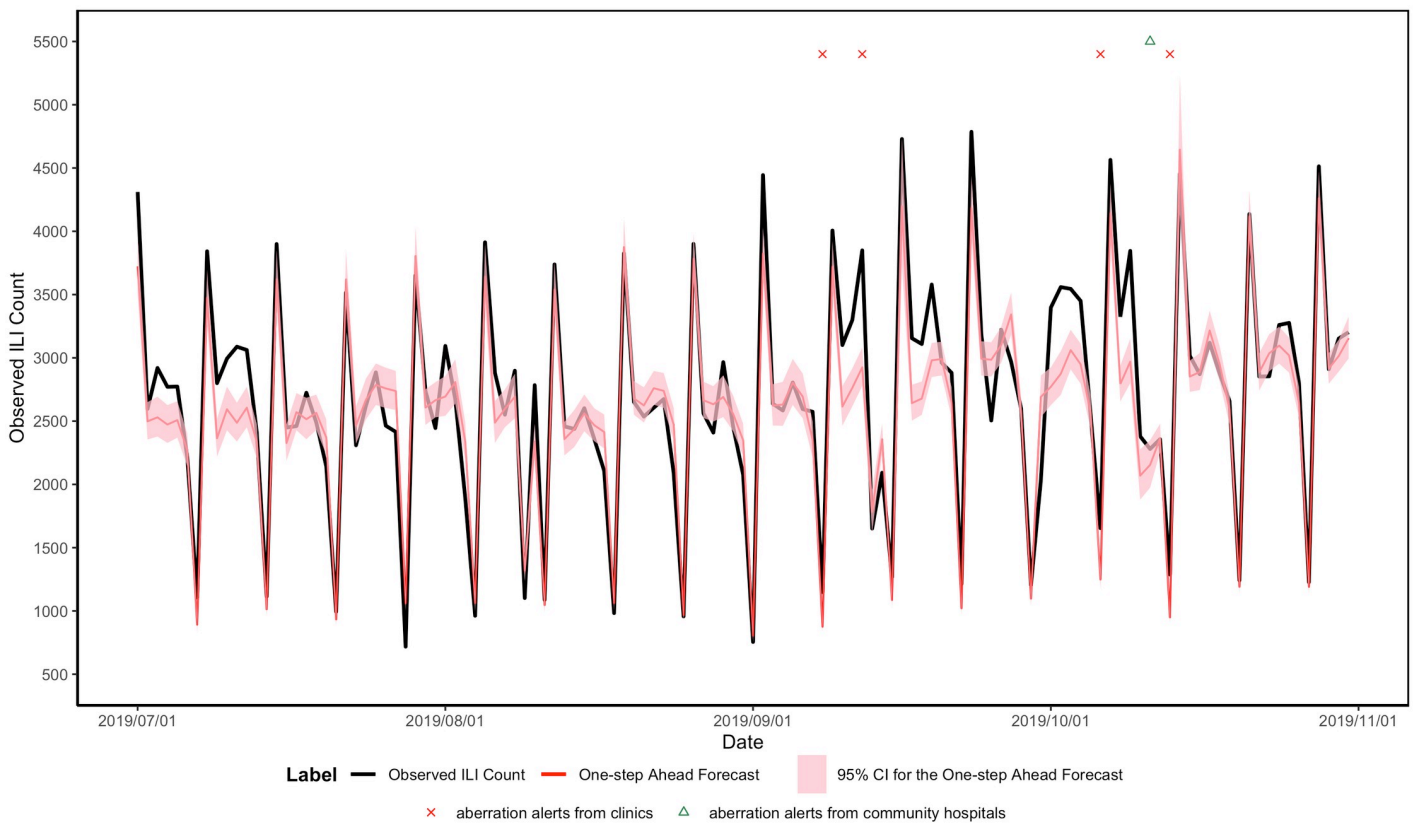
One-step-ahead forecasts for forecast period for ILI syndromes in a rolling manner.

**Fig 5:**
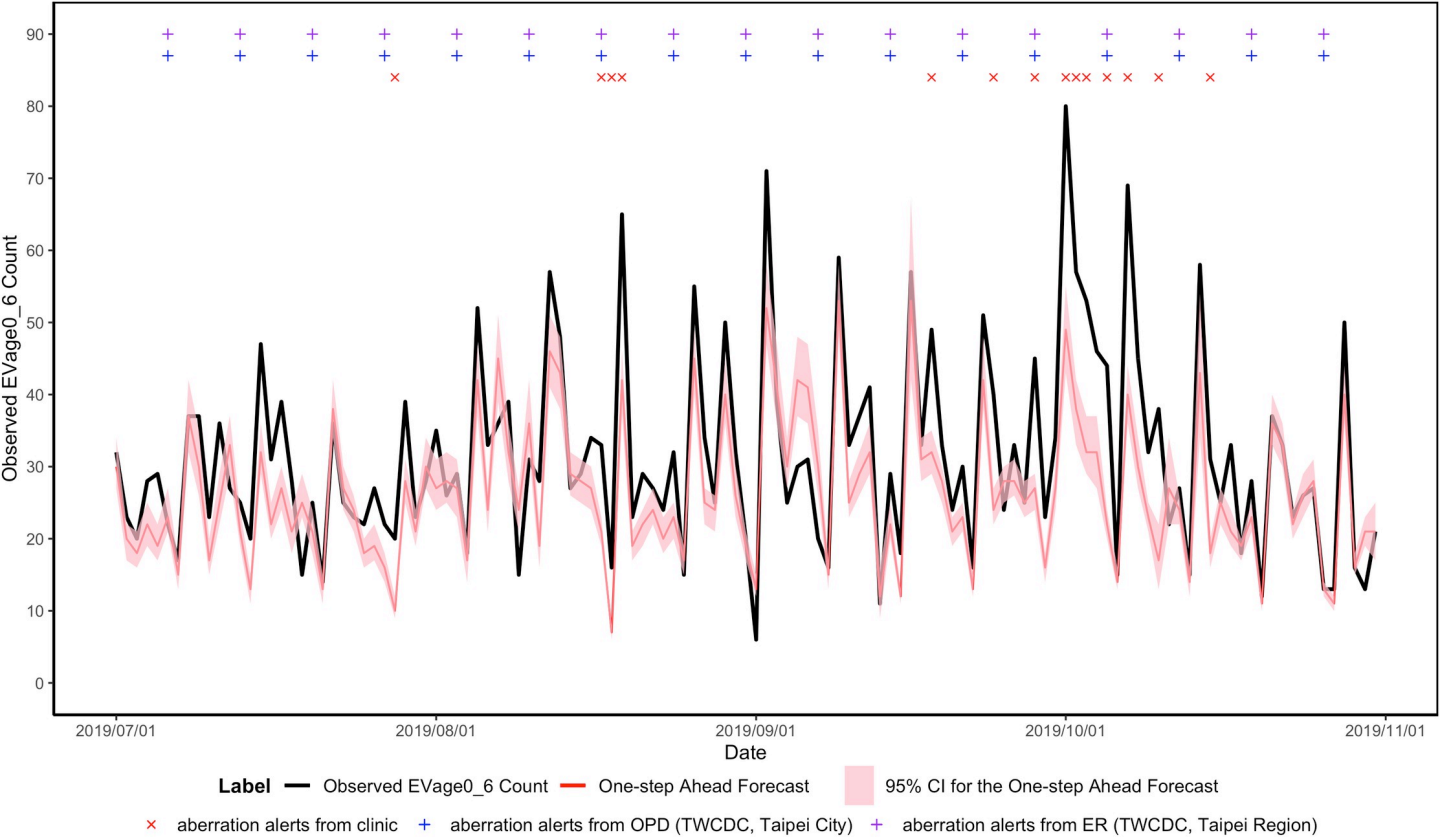
One-step-ahead forecasts for forecast period for EV syndromes in a rolling manner.

The one-step-ahead forecasts for ILI and EV syndromes are shown in Figs 4 and 5, respectively. The overall predicted ICD counts pattern was consistent with the observed ICD counts pattern. The RMSEs of ILI and EV are 308.78 and 9.27. The MAPEs of ILI and EV are 0.0917 and 0.2161.

## Discussion

Syndromic surveillance in ER settings around the world has shown good performance on early warning of infectious diseases [23, 24]. On the other hand, sentinel physician surveillance in communities can capture milder symptoms and is better suited to a younger population, including pre-school children, students and young adults. With the help of health informatics, the traditional way to report diseases manually by physicians can be replaced with an automatic approach to compute the matching ICD-10 codes of the specific syndromes from hospital information systems. This method does not change the behavior of the physicians, i.e. involves no additional workload, and could reduce the potential recall bias and improve the timeliness of surveillance. This study demonstrates that applying the concept of syndromic surveillance in clinics is feasible and performs well for aberration detection. Clinics are the fundamental primary care system in communities and can reflect residents’ health condition locally. Medical practitioners participating in the Sentinel plus system can access the system to obtain aggregated results of the prevalence of symptoms in neighboring areas. They also can view the aberration signal at the city level and also can check the risk level of their own clinic and their surrounding areas. This system not only provides warning signals to the local health department for managing the risks but also reminds the medical practitioners to be vigilant toward susceptible patients.

The two syndromic groups were used to develop methods for improving the temporal accuracy of early aberration detection in this study. However, a hospital-emergency-room-based real-time outbreak and disease surveillance system has been established by Taiwan’s CDC, which aims to collect individual ICD-9-CM and ICD-10 codes from patient ER visits at designated emergency response hospitals. A week later, a surveillance report is released by the Taiwan CDC’s open data portal. The focuses of the ER system and Sentinel plus are different. Sentinel plus focuses on mild diseases at the early stage of an epidemic in a community, whereas the ER system focuses on severe diseases, and the patients might come from a wider area, including other townships and counties.

A growing body of epidemiological and clinical evidence has shown that daily air pollution and meteorological factors increase the risks of numerous diseases [19–22]. In this study, environmental air pollutants such as PM_2.5_, PM_10_, ozone, nitrogen oxides, sulfur dioxide and carbon monoxide and meteorological factors, such as daily maximum, minimum, and average temperatures, relative humidity, air pressure, wind speed, and precipitation, were considered as covariates for model building. Our study suggested that daily average temperatures, relative humidity, ozone, and PM_2.5_ were associated with ILI cases, and daily minimum temperatures, relative humidity, ozone, and PM_2.5_ were associated with EV cases.

Model selection is an important part of any statistical analysis. In this study, the model is selected based on hypothesis testing. Final estimation, interpretation and prediction are then based on the selected model. We used a negative binomial regression model to couple with lag structure functions to examine the short-term association between ICD counts and meteorological variables. Historically, a primary use of regression was to illuminate a supposed relationship between independent variables and an outcome variable. The goal has been to understand an important relationship and explain it using the data that the regression was fit to. This step does not directly address predictive accuracy, but it can provide useful insight in a predictive setting. With the advent of big data, regression is widely used to form a model to predict individual outcomes for new data, rather than explain data in hand (i.e., a predictive model). Variable selection methods are used to reduce dimensionality and create more compact models. In this case, the focus is not on predicting individual cases, but rather on understanding the overall relationship. Statistical tests can help us to investigate the statistical significance of the relationships modelled through regression analysis; however, some limitations should be noticed. Results of significance tests are based on probabilities and as such cannot be expressed with full certainty. When a test shows that a difference is statistically significant, then it simply suggests that the difference is probably not due to chance. Statistical inferences based on the significance tests cannot be said to be entirely correct evidence concerning the truth of the hypothesis. This problem occurs because all hypothesis tests have a false discovery rate. Thus we should avoid misinterpretations and misuses of statistical significance tests.

We used Pearson residuals of the proposed GLM model for detecting aberrations of an epidemic in the early stage, which is that if the Pearson residual per day in an area exceeds a critical value (say 1.96, in this study), an epidemic in that area will begin in the following weeks (a warning of an epidemic). For the two syndromic groups illustrated in this study, the early stages of an epidemic might be detectable in pre-epidemic periods. The alert signals of the proposed methods appeared earlier than those of OPD and ER surveillance. There were still sporadic signals during post-epidemic periods, because ICD-10 counts were higher than in the non-epidemic periods. The epidemic aberrations presented herein are based on ICD-10 counts of syndromic groups from clinics. Because clinics were recruited on a voluntary basis, our results might include potential biases. For building a stable surveillance system and applying the proposed method of epidemic warning to surveillance, it will be important to further study setting thresholds for the onset and end of epidemics for specific diseases.

In our model performance, data with low counts have an increased risk of producing false positive alarms. This can be solved by setting the threshold value, which is determined by the best compromise between sensitivity and specificity for a given disease. However, a higher threshold will cause a lower sensitivity. Thus, another solution is as follows. If aberrations are significant on consecutive days, then an aberration alert is issued. We expect this rule will reduce the number of false positive alarms without reducing the system sensitivity.

Influential points can seriously distort all aspects of data analysis such as altering the estimation of the regression coefficient and swaying the outcome of statistical inference. Detecting multiple influential observations is much more challenging in a high-dimensional setting, due to the notorious “masking” and “swamping” effects. In the language of multiple testing, masking is the problem of getting false negatives and swamping is the problem of getting false positives. In this paper, the masking effect would not have substantially influenced our results by examining forecasting performance. However, we might encounter this problem in the future for other symptom groups surveillance. To quantify the influence of an observation, we can compare a predefined measure evaluated on the whole dataset and the measure evaluated on a subset of the data leaving out the observation under investigation. However, multiple influential observations are commonly encountered, and measures based on the leave-one-out approach may be ineffective when there are multiple influential observations due to the masking and swamping effects. Since the number of influential observations is generally unknown in practice, it is natural to employ a notion of leave-many-out or group deletion.

Routine analysis of public health surveillance data to detect departures from historical patterns of disease frequency is required to enable timely public health responses to decrease unnecessary morbidity and mortality. The aim of such surveillance includes detection of epidemics, especially detection in the early stage, essential for the control of epidemics of infectious diseases. Because infectious disease threats usually start locally and subsequently spread widely, observation of the data for small areas is important in order to deny them the opportunity to spread further among people and overwhelm health systems. For example, Weng et al. [25] observed by using spatio-temporal analysis that the epidemic patterns of EV and ILI both diffuse from the northern suburban districts to central Taipei. Chan et al. [26] found that mild EV cases had begun to rise in May 2008, and the outbreak spread from south to north before the detected spatio-temporal clusters in June 2008. Chan et al. [8] found that the 2009 H1N1 pandemic flu in different regions of Taiwan reflected different waves of transmission in August 2009. Tang et al. [27] proposed that the influence of latitude variation on the spatial spreading of HFMD provided an important basis for detecting HFMD epidemic trends. Thus, warnings of epidemics in small areas might provide information on epidemics in large areas. Our system not only provides warning signals to the local health department for managing the risks but also reminds medical practitioners to be vigilant toward susceptible patients. Participating clinics can view aberration signals at the city level and also can check the risk level of their own clinic and neighboring areas. Although a national surveillance system can provide nationwide data, it takes time to integrate information from all over the country. For government decision making, it is helpful to have predictions of future epidemic trends. For this purpose, our surveillance system can provide both high spatial and temporal resolution for understanding the progress of an epidemic.

In this study, we concurrently compared the signals of ILI in clinics and community hospitals. The results found that the signals detected from clinics were earlier than the signals detected from outpatient surveillance, from ER surveillance of Taiwan’s CDC, and also from community hospitals. In addition, we found that the signals from outpatient surveillance of Taiwan’s CDC were also earlier than community hospitals’. The community hospitals’ signals were only earlier than ER surveillance of Taiwan’s CDC. The data from outpatient surveillance were originally from national health insurance data including different levels of care providers, from clinics to large hospitals. Although the total visiting volume was quite high in outpatient surveillance, the patients of medical centers and regional hospitals were mostly from other cities or townships because of the pattern of healthcare-seeking behaviors [28]. This might dilute the true epidemic situation locally. In addition, the temporal resolution of outpatient surveillance was weekly and the spatial resolution was at the city level. However, the clinic surveillance can have better daily temporal resolution and village (community) spatial resolution. ER surveillance here reflects the peaking wave of the ILI epidemic during Chinese New Year vacation, when most of hospitals and clinics were closed, and only ERs were open [29].

Statistical process control (SPC) combines time series analysis methods with graphical presentation of data, often yielding insights into the data more quickly and in a way more understandable to lay decision makers. Its primary tool, the control chart, provides us with a method of better understanding data for monitoring epidemic disease outbreaks. Although SPC is a versatile tool which can help us identify long-term and consistent change in trends, it usually cannot identify rapid outbreaks. A comparison to more traditional charts such as the u-chart and c-chart would be more relevant.

In addition, we also compared a statistical process control method, cumulative sum (CUSUM), and our proposed method. CUSUM [30, 31] is used widely in surveillance systems because it is easy to compute and interpret. Results of aberration signals for ILI and EV-like syndromes generated by the proposed method and CUSUM are shown in S4 and S5 Files, respectively. In terms of early aberration detection performance, our proposed method outperforms CUSUM. The latter tends to detect sustained changes, because it accumulates the deviations from the mean value over an interval rather than considering deviations at a single time point. Because of its cumulative nature, it may miss subtle changes in the early stages of the outbreak that occur leading up to a major increase in disease incidence. This feature also causes warning signals to continue to appear after the epidemic. In contrast, the alarm signals of our proposed method can detect the subtle changes in the early stage of the outbreak.

The annual epidemic seasons of enterovirus in Taiwan were May to June and September to October [26, 32]. In our study period, we indeed detected EV-like signals among those aged from 0 to 6 in September 2018 and May-June 2019. However, we also detected another big wave of EV epidemic in November-December 2018. In clinics, we found the first signal was issued on November 17, 2018. The next week (the 48^th^ week of 2018), Taiwan CDC issued the EV weekly report indicating the EV isolation rate in communities was 47.8% (https://www.cdc.gov.tw/Category/MPage/0IaI1fGlb_ZPJ_ER_aJWKg). From the 49^th^ week to 52^th^ week of 2018, the EV isolation rates were 60%, 43.8%, 41.3% and 54.9%, respectively. The major subtype of EV was Coxsackie A virus, exceeding 80% of all positive isolates. The signals from clinics were earlier than outpatient and ER surveillance of Taiwan’s CDC. We found that the patients aged 0–6 accounted for 21% of total visits in clinics, but the same age group in community hospitals only accounted for 4%. That might be good evidence of the need to monitor pediatric-related infections in clinics.

### Limitations

Several limitations of this study should be mentioned. First, the enhanced clinic-based sentinel surveillance system, Sentinel plus, started to recruit the clinics in June 2018, but it has only been slightly over a year since data collection began for evaluation of the system in this study, and this may limit the generalizability of the study results. However, the recruiting process is still ongoing, and we believe that an increase in the number of clinics will help us capture more early-alert signals. Second, the system does not include identifiers for individual patients, but instead collects syndromes diagnosed and ICD codes automatically fetched by the hospital information systems. Therefore, some patients who made visits more than once may have had their visits counted independently, yielding inaccurate estimates of variance. Because the system included at most five diagnosis codes for each patient, and it is less likely that patients would visit medical care providers more than once in the same day, this limitation might affect our conclusion only to a small degree. Third, lag structures and model coefficients are not fixed over time. Thus, the model should be established and calibrated according to the different characteristics of infectious diseases and the varied needs of different areas. The incorporation of automatic model calibration should be considered in further studies.

## Conclusions

Traditional surveillance systems often operate with considerable delay; thus complementary surveillance systems are required to provide the necessary lead time. Syndromic surveillance systems may fulfil this role. Clinics are the fundamental primary care system in communities and can reflect the residents’ health condition locally. The Sentinel plus system is the fundamental primary surveillance system in communities and can reflect the residents’ health condition locally. Medical practitioners participating in the system can access the system to obtain aggregated results of the prevalence of symptoms in neighboring areas. This system not only provides warning signals to the local health department for managing the risks but also reminds the medical practitioners to be vigilant toward susceptible patients. The innovations of this study are to set up the community-based syndromic surveillance system and to consider the simplicity and stability of the statistical model for routine surveillance to provide timely signals. We are convinced that the temporal and spatial resolution of this system is better than traditional symptom monitoring systems. The near real-time surveillance can help decision makers take action or evaluate policies for controlling epidemics.

## Supporting information

S1 File List of 23 syndromic groups in Sentinel plus. (PDF)

S2 File Snapshot of Sentinel plus. The base map tile is from OpenStreetMap and OpenStreetMap Foundation.
(PDF)

S3 File Multicollinearity diagnostics by variance inflation factor (VIF) for explanatory variables in the final model. (PDF)

S4 File Results of aberration signals for ILI syndrome generated by the proposed method and CUSUM. (TIF)

S5 File Results of aberration signals for EV-like syndrome generated by the proposed method and CUSUM. (TIF)
