# Supplementary material for: Approaching precision public health by automated syndromic surveillance in communities
Source: PLoS One. 2021 Aug 6;16(8):e0254479. doi: 10.1371/journal.pone.0254479 (PMC8345830; doi:10.1371/journal.pone.0254479)
Supplement: S1 File — (PDF) [file pone.0254479.s001.pdf]

S1 File. The list of 23 syndromic groups in Sentinel plus

1. Influenza-like illness (ILI)
2. Enterovirus-like illness (EV-like)
3. Dengue-like illness
4. Dengue fever
5. Chikungunya
6. Fever
7. Influenza
8. Pneumonia
9. Acute upper respiratory infection
10. Respiratory syncytial virus (RSV)
11. Diarrhea
12. Respiratory diseases
13. Acute bronchitis
14. Asthma
15. Cough
16. Gastrointestinal–lower
17. Gastrointestinal–upper
18. Gastrointestinal hemorrhage
19. Foodborne illness
20. Rotavirus
21. Rash
22. Skin infection
23. Urinary tract infection
