# Supplementary material for: Approaching precision public health by automated syndromic surveillance in communities
Source: PLoS One. 2021 Aug 6;16(8):e0254479. doi: 10.1371/journal.pone.0254479 (PMC8345830; doi:10.1371/journal.pone.0254479)
Supplement: S2 File — The base map tile is from OpenStreetMap and OpenStreetMap Foundation. (PDF) [file pone.0254479.s002.pdf]

S2 File. The snapshot of Sentinel plus.

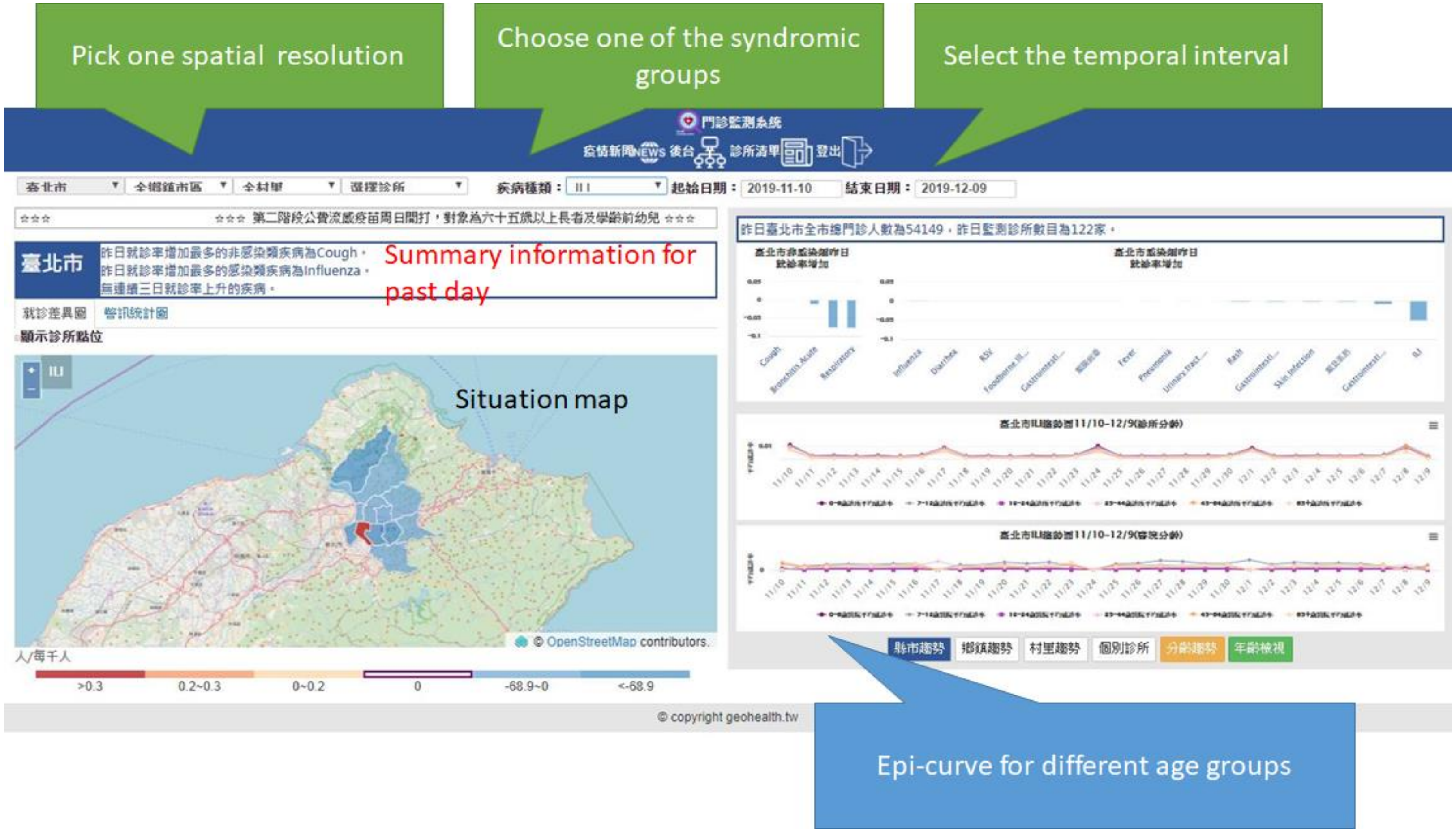

*The base map tile is from OpenStreetMap and OpenStreetMap Foundation.
