# Supplementary material for: Approaching precision public health by automated syndromic surveillance in communities
Source: PLoS One. 2021 Aug 6;16(8):e0254479. doi: 10.1371/journal.pone.0254479 (PMC8345830; doi:10.1371/journal.pone.0254479)
Supplement: S3 File — (PDF) [file pone.0254479.s003.pdf]

S3 File. Multicollinearity diagnostics by variance inflation factor (VIF) for explanatory variables in final model

		ILI	EV
Day of the week			
	Monday	1.759	1.991
	Tuesday	1.723	1.771
	Wednesday	1.735	1.768
	Thursday	1.738	1.775
	Friday	1.730	1.786
	Saturday	-	-
	Sunday	1.697	1.491
Public holiday			
	Public holiday	2.777	2.157
	Day after public holiday	-	-
	Days excluding both public holidays and day after public holidays	2.829	2.189
Temperature (daily average)		1.095	-
Temperature (daily minimum)		-	1.092
Relative humidity		1.104	1.196
O_3		1.227	1.189
$PM_{2.5}$		1.198	1.179
