# Supplementary figures and images for: Approaching precision public health by automated syndromic surveillance in communities
Source: PLoS One. 2021 Aug 6;16(8):e0254479. doi: 10.1371/journal.pone.0254479 (PMC8345830; doi:10.1371/journal.pone.0254479)

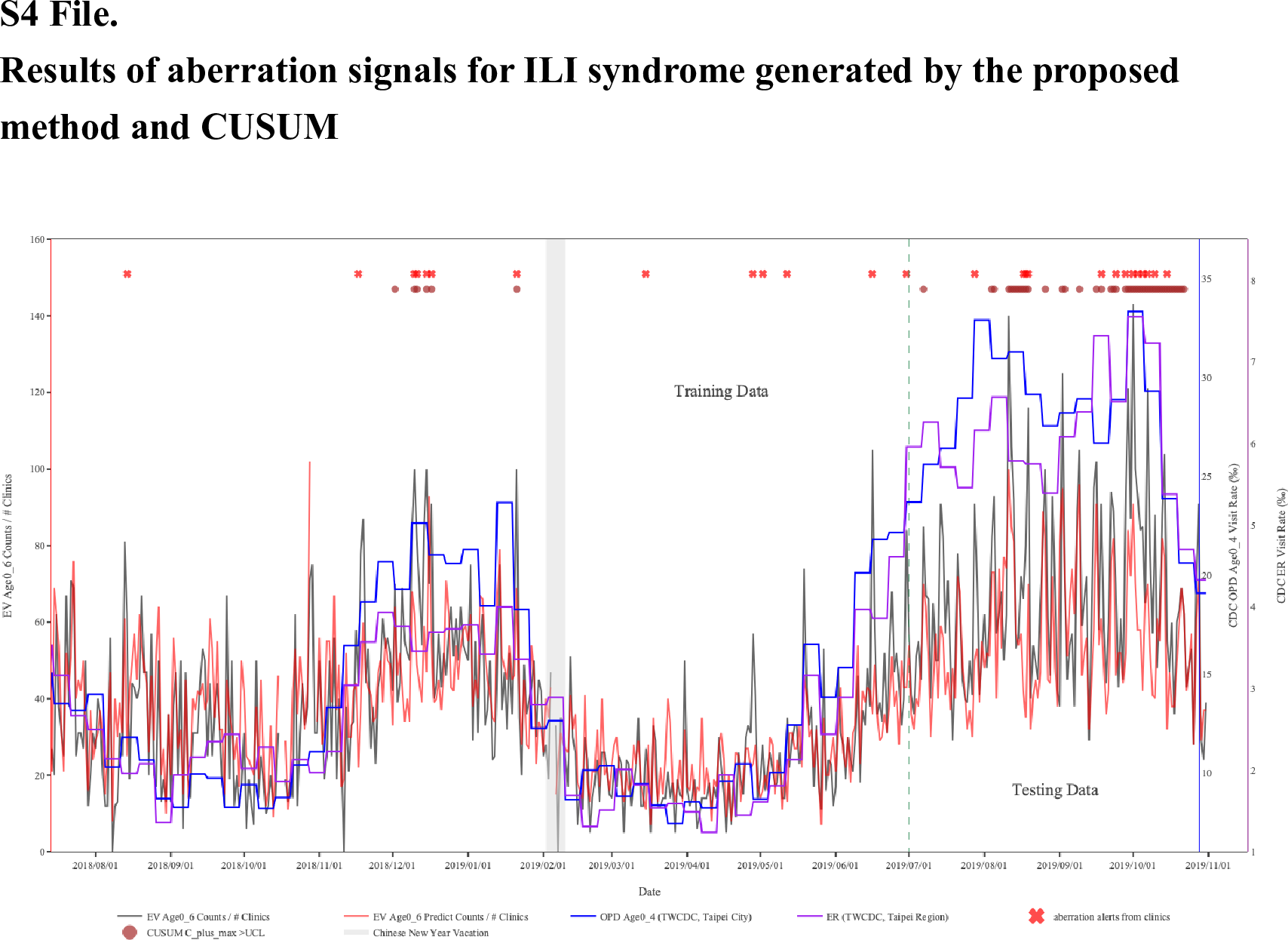

Supplement: S4 File — (TIF) [file pone.0254479.s004.tif]

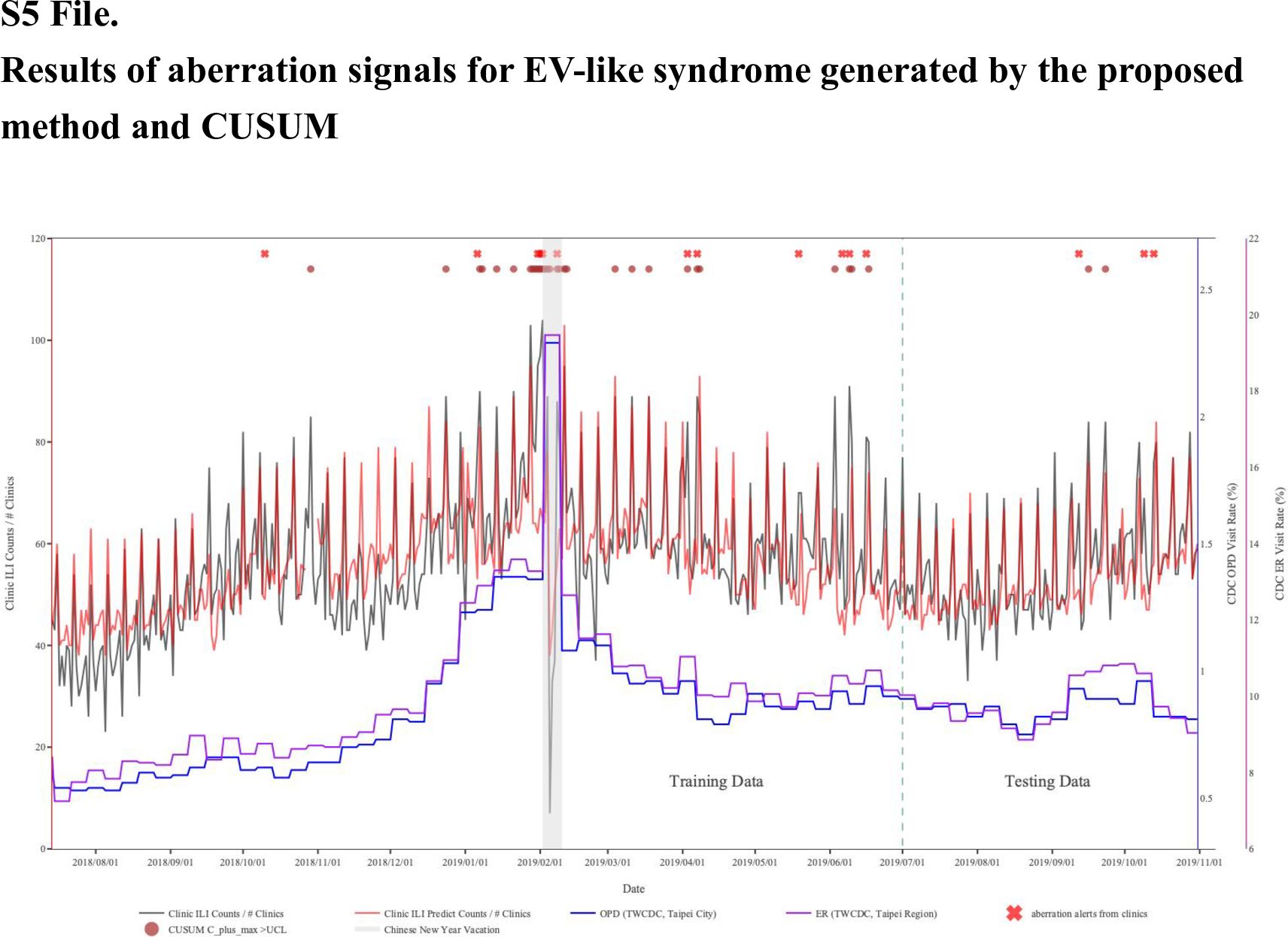

Supplement: S5 File — (TIF) [file pone.0254479.s005.tif]
